# An exploration of the increasing prevalence of chronic pain among Canadian veterans: Life After Service Studies 2016 and 2019

**DOI:** 10.1080/24740527.2024.2443631

**Published:** 2025-01-30

**Authors:** Jhalok Ronjan Talukdar, Dena Zeraatkar, Andrew Thomas, Jason W. Busse

**Affiliations:** a Department of Anesthesia, McMaster University, Hamilton, Ontario, Canada; b Department of Health Research Methods, Evidence, and Impact, McMaster University, Hamilton, Ontario, Canada; c Canadian Armed Forces Health Services Centre, Edmonton, Alberta, Canada; d Michael G DeGroote National Pain Centre, McMaster University, Hamilton, Ontario, Canada

**Keywords:** Chronic pain, Life After Service Study, chronic pain among Canadian Veterans, Canadian Armed Forces, cross-sectional survey

## Abstract

**Background:**

The Life After Service Study (LASS) suggests that the absolute prevalence of chronic pain among Canadian veterans, defined as pain lasting 3 months or longer, increased by 10% from 2016 to 2019.

**Aims:**

We explored the association of year of survey administration, sociodemographic characteristics, military service, and health-related factors with the prevalence of chronic pain among Canadian veterans.

**Methods:**

We analyzed 2016 and 2019 LASS data and built a multivariable regression model to explore factors associated with chronic pain. Measures of association are reported as adjusted odds ratios (ORs) and absolute risk increases (ARIs).

**Results:**

The 2016 LASS (73% response rate; 3002 of 4121) reported a 41.4% prevalence of chronic pain, and the 2019 LASS (72% response rate; 2630 of 3671) reported a 51.5% prevalence of chronic pain among Canadian veterans. Respondents who completed the 2019 LASS were more likely to endorse an anxiety or related disorder, mood disorder, probable posttraumatic stress disorder, and traumatic brain injury. In our adjusted regression model, year of survey administration was not associated with chronic pain (OR = 1.08, *P* = 0.8); however, we found large associations with obesity class 1 (body mass index [BMI] = 30.0–34.9; OR = 3.66; 95% confidence interval [CI] 1.46–9.17; ARI 27%), obesity class 2 (BMI = 35.0–39.9; OR = 8.10; 95% CI 1.67–39.3; ARI 47%), mood disorder (OR = 3.20; 95% CI 1.49–6.88; ARI 24%), and an anxiety or related disorder (OR = 4.53; 95% CI 1.28–16.0; ARI 33%).

**Conclusions:**

The increase in chronic pain among Canadian veterans from 2016 to 2019 appears confounded by increased comorbidities associated with chronic pain among responders in 2019.

## RÉSUMÉ

**Contexte :** L’étude Life After Service Study (LASS) indique que la prévalence absolue de la douleur chronique chez les anciens combattants canadiens, définie comme une douleur d’une durée de trois mois ou plus, a augmenté de 10 % entre 2016 et 2019.

**Objectifs :** Nous avons étudié l’association entre l’année de réalisation de l’enquête, les caractéristiques sociodémographiques, le service militaire et les facteurs liés à la santé, et la prévalence de la douleur chronique chez les anciens combattants canadiens.

**Méthodes :** Nous avons analysé les données de l’étude LASS de 2016 et 2019 et construit un modèle de régression multivariable afin d’explorer les facteurs associés à la douleur chronique. Les mesures d’association sont présentées sous forme de rapports de cotes ajustés (RC) et d’augmentations absolues du risque (AAR).

**Résultats :** L’enquête LASS de 2016 (taux de réponse de 73 %; 3002 répondants sur 4121) a fait état d’une prévalence de 41,4 % de la douleur chronique, tandis que l’enquête LASS de 2019 (taux de réponse de 72 %; 2 630 répondants sur 3 671) a révélé une prévalence de 51,5 % de la douleur chronique chez les anciens combattants canadiens. Les répondants à l’enquête LASS de 2019 étaient plus susceptibles de souffrir d’anxiété ou de troubles connexes, de troubles de l’humeur, de troubles de stress post-traumatique probables et de lésions cérébrales traumatiques. Dans notre modèle de régression ajusté, l’année de réalisation de l’enquête n’était pas associée à la douleur chronique (RC = 1,08, P = 0,8). Toutefois, nous avons trouvé des associations importantes avec la classe d’obésité 1 (indice de masse corporelle [IMC] = 30,0 - 34,9; RC = 3,66; intervalle de confiance à 95 % [IC] 1,46 - 9,17; AAR de 27 %), la classe d’obésité 2 (IMC = 35,0 - 39,9 ; RC = 8,10; IC à 95 % : 1,67-39,3; AAR de 47 %), les troubles de l’humeur (RC = 3,20 ; IC à 95 % : 1,49-6,88; AAR de 24%) et un trouble anxieux ou apparenté (RC = 4,53; IC à 95% : 1,28 - 16,0; AAR de 33%).

**Conclusions :** L’augmentation de la douleur chronique chez les anciens combattants canadiens entre 2016 et 2019 semble être confondue par l’augmentation des comorbidités associées à la douleur chronique chez les répondants de 2019.

## Introduction

Chronic pain, which persists beyond the normal time required for healing and is commonly defined as pain lasting ≥3 months, impacts approximately 20% of the general population in Canada, the United States, and globally.^1–7^ In Canada, two-thirds of people living with chronic pain experience moderate to severe symptoms, and half report living with chronic pain for over a decade.^2^ Chronic pain is not evenly distributed among the Canadian population, with a higher prevalence reported among older adults, Indigenous peoples, women, veterans, and people who use drugs.^2^

Chronic pain affects veterans’ well-being, with 54% reporting pain interference with work and 77% experiencing life stress most days.^8^ There is also a strong association between chronic pain and mental health, and 63% of veterans living with chronic pain meet diagnostic criteria for a mental illness.^8^ Several factors may contribute to the higher rates of chronic pain among veterans. These include the physically demanding nature of military service, a higher prevalence of mental health conditions compared to the general population, combat-related injuries, potential delays in accessing appropriate pain management during the transition from military to civilian health care systems, and cultural factors such as military emphasis on resilience that may lead to delayed reporting of pain.^8–12^

The Life After Service Study (LASS), conducted by Statistics Canada every 3 years, is a national survey that collects information from military veterans. According to results from the LASS, the percentage of Canadian veterans reporting chronic pain increased by 10.1% over the two most recent administrations, from 41.4% in 2016^6^ to 51.5% in 2019.^13^ The underlying factors for this increase remain unclear; however, we hypothesized that contributing factors may include older age or confounding by other factors associated with chronic pain. Specifically, veterans who responded to the LASS in 2019 may have been systematically different than those who responded in 2016.

## Materials and methods

### Study design and participants

The LASS is a national cross-sectional telephone survey that collects comprehensive data on Canadian Armed Forces (CAF) Regular Force veterans, including their health, well-being, and demographic factors.^14^ The 2016 LASS surveyed veterans discharged from the CAF between 1998 and 2015, and the 2019 LASS surveyed veterans discharged between 1998 and 2018. Eligible respondents for both surveys were CAF members who were not currently enlisted, resided in Canada’s ten provinces, were not in a long-term care facility, and did not hold entry rank at discharge.^14,15^ We included 3002 participants (73% response rate; 3002 of 4121)^6^ from the 2016 LASS (representing a weighted total population of 56,413) and 2630 participants (72% response rate; 2630 of 3671)^16^ from the 2019 LASS (representing a weighted total population of 63,948) in our analysis. The same veterans could potentially participate in multiple cycles of the LASS; however, the data do not allow for identification of common respondents between administrations.

### Variables

#### Chronic pain

We classified participants as having chronic pain if they negatively endorsed the following item on the 2016 or 2019 LASS survey: “Are you usually free of pain or discomfort?” (yes or no). Though the descriptor “usually” implies a temporal aspect, it is not clear whether veterans who responded negatively to this question had experienced pain for 3 months or longer. Nevertheless, we accepted this definition of chronic pain to maintain consistency with previous analyses of LASS data^6,16^ reporting on the prevalence of chronic pain, to facilitate direct comparisons.^6,13^ If a respondent endorsed living with chronic pain on the LASS, then pain intensity and activity limitations were captured through the following questions: (1) “How would you describe the usual intensity of your pain or discomfort?” (mild, moderate, or severe) and (2) “How many activities does your pain or discomfort prevent?” (none, a few, some, or most).

#### Covariates

We captured data on 11 covariates to include in our regression model: age at the time of survey completion, sex (male or female), formal education (less than high school graduation, high school graduation, postsecondary graduation, or university graduation), annual household income (<$50,000 or ≥$50,000), military rank at discharge (junior noncommissioned member [NCM], officer, or senior NCM), military environment (sea, land, or air), body mass index (BMI; underweight: BMI < 18.5, normal: BMI = 18.5–24.9, pre-obese: BMI = 25.0–29.9, obese class 1: BMI = 30.0–34.9, obese class 2: BMI = 35.0–39.9, obese class 3: BMI ≥ 40), alcohol consumption (every day, weekly, monthly), self-reported mood disorder (e.g., major depressive disorder, persistent depressive disorder, bipolar disorder; yes or no), an anxiety or related disorder (e.g., phobia, obsessive compulsive disorder, panic disorder; yes or no), and probable posttraumatic stress disorder (PTSD; yes or no). Regarding mental health conditions, the LASS assesses anxiety or related disorders and mood disorders by asking participants whether they are experiencing each of these conditions. These questions specifically inquire about conditions diagnosed by a health professional that are expected to last, or that have already lasted, 6 months or more. The LASS assesses probable PTSD if respondents endorse at least three of four questions about this disorder (see Table A1).

Our selection of covariates was informed by prior research demonstrating positive associations between older age, female sex, higher BMI, mental illness, lower formal education attainment, and lower household income with chronic pain.^11,17–19^ Lower military rank may entail greater exposure to physical and psychological stressors, which can increase the risk of chronic pain.^19,20^ Similarly, military environment (sea, land, or air) may influence the development of chronic pain, with land-based roles often involving more physical strain and higher risk of injury.^21^ Further, a 2022 systematic review found that any alcohol consumption was associated with a 24% lower odds of reporting chronic pain.^22^

### Statistical analysis

We present descriptive statistics to summarize the characteristics of participants and the distribution of chronic pain among Canadian veterans between surveys, using weighted estimations. We report categorical variables as frequencies and percentages and continuous variables as means and standard deviations (SDs) when normally distributed and as medians with interquartile ranges otherwise. We used the Wilcoxon rank sum test for complex survey samples for continuous variables and the chi-square test with Rao and Scott’s second-order correction, accounting for survey design, for categorical variables, to compare responses between 2016 and 2019. Bootstrap weights provided by Statistics Canada were applied to convert unweighted frequencies to represent the Canadian veteran population.

Using weighted data from both the 2016 and 2019 LASS surveys, we constructed a multivariable logistic regression model to identify factors associated with chronic pain among Canadian veterans. Our independent factors were (1) year of survey administration, (2) age, (3) sex, (4) formal education, (5) household income, (6) military rank, (7) military environment, (8) BMI, (9) alcohol consumption, (10) self-reported mood disorder, (11) self-reported anxiety or a related disorder, and (12) probable PTSD. To avoid overfitting, we required ten or more events per variable included in our regression model.^23^ We excluded response options that were endorsed by less than 3% of respondents. We reported relative measures of association as odds ratios (ORs) and 95% confidence intervals (95% CIs). Multicollinearity was deemed concerning if the variance inflation factor for any independent variable was greater than five.^24^ All comparisons were two-tailed, and an independent factor was considered statistically significant if it had a *P* value <0.05 in our multivariable model. For all statistically significant factors, we calculated the absolute risk increase (ARI) to optimize interpretability. We established the baseline risk for reporting chronic pain by calculating the prevalence among those veteran respondents without statistically significant risk factors for chronic pain. We used the Hosmer-Lemeshow test^25^ to assess the goodness of fit of our logistic regression model, and conducted all analyses with R v4.3.3.^26^

## Results

Respondents to the 2016 and 2019 LASS surveys were similar in sex distribution, BMI, marital status, household income, military rank, and environment. Approximately 87% of veterans were men, 62% were married, more than 80% reported household incomes of ≥$50,000 per year, and most held a junior NCM military rank at discharge. Respondents in 2019 were an average of 2.2 years older (SD 0.07), more likely to have completed postsecondary education (41% vs. 36%), and less likely to be employed (59% vs. 66%) than respondents in 2016 (Table 1).

**Table 1.** Characteristics of participants.

Variables	LASS 2016 (weighted *n* = 56,413)	LASS 2019 (weighted n = 63,948)	*P* value^a^
**Age, mean (SD)**	47.9 (11.7)	50.1 (11.8)	<0.001
**Sex**			0.6
Male	49,529 (87.8%)	55,667 (87.2%)	
Female	6,885 (12.2%)	8,162 (12.8%)	
**BMI**			0.1
Underweight	297 (0.5%)	299 (0.5%)	
Normal weight	13,503 (24.2%)	14,731 (23.3%)	
Pre-obese	25,674 (45.5%)	26,917 (42.1%)	
Obese class 1	11,263 (20.2%)	14,978 (23.7%)	
Obese class 2	3,726 (6.7%)	4,520 (7.1%)	
Obese class 3	1,295 (2.3%)	1,847 (2.9%)	
**Education**			0.01
University graduation	9,510 (17.2%)	11,376 (18.2%)	
High school graduation	23,322 (42.3%)	23,007 (36.7%)	
Less than high school graduation	2,294 (4.2%)	2,820 (4.5%)	
Postsecondary graduation	20,029 (36.3%)	25,473 (40.6%)	
**Marital status**			0.6
Divorced	3,912 (6.9%)	4,681 (7.3%)	
Living common-law	8,400 (14.9%)	9,485 (14.8%)	
Married	34,732 (61.6%)	40,234 (63.0%)	
Separated	1,863 (3.3%)	1,923 (3.0%)	
Single, never married	7,077 (12.6%)	6,917 (10.8%)	
Widowed	406 (0.7%)	668 (1.1%)	
**Employment status**			<0.001
Employed	36,435 (66.1%)	36,882 (59.1%)	
Disabled	4,742 (8.6%)	7,466 (12.0%)	
Retired	9,081 (16.5%)	13,409 (21.5%)	
Unemployed	4,904 (8.9%)	4,632 (7.4%)	
**Household income**			0.5
<50,000	771 (20.4%)	814 (16.4%)	
≥50,000	3,013 (79.6%)	4,141 (83.6%)	
**Military rank**			>0.9
Junior NCM	29,429 (52.2%)	33,465 (52.3%)	
Officer	9,768 (17.3%)	11,149 (17.4%)	
Senior NCM	17,217 (30.5%)	19,334 (30.2%)	
**Military environment**			0.4
Sea	10,010 (17.7%)	10,420 (16.3%)	
Land	29,214 (51.8%)	33,052 (51.7%)	
Air	17,190 (30.5%)	20,476 (32.0%)	

^a^Wilcoxon rank sum test for complex survey samples; chi-square test with Rao and Scott’s second-order correction.

Compared to 2016, veterans who completed the LASS in 2019 were more likely to report chronic pain (41.4% vs. 51.5%); pain severity and activity limitations among those with chronic pain were similar between administrations. Consistent with the higher prevalence of chronic pain, respondents to the 2019 LASS also reported higher prevalences of pain-related conditions, including back problems (46% vs. 41%), arthritis (35% vs. 30%), and migraine headaches (17% vs. 14%). Veterans who completed the 2019 LASS were also more likely to self-report an anxiety or related disorder (22% vs. 15%), a mood disorder (26% vs. 21%), probable PTSD (25% vs. 17%), and traumatic brain injury (7% vs. 4%; Table 2).

**Table 2.** Chronic pain characteristics and other comorbidities.

Variables	LASS 2016 (weighted *n* = 56,413), *n* (%)	LASS 2019 (weighted *n* = 63,948), *n* (%)	*P* value^a^
**Chronic pain**	23,350 (41.4)	32,802 (51.5)	<0.001
**Pain intensity**			0.8
A few	14,190 (61.0)	19,837 (60.6)	
None	5,124 (22.0)	7,007 (21.4)	
Some	3,933 (16.9)	5,891 (18.0)	
**Activity prevention**			0.3
Mild	2,671 (16.7)	3,014 (13.5)	
Moderate	5,771 (36.2)	8,764 (39.2)	
Severe	7,516 (47.1)	10,561 (47.3)	
**Back problem**	23,104 (41.0)	29,181 (45.8)	0.01
**Arthritis**	16,739 (29.7)	22,503 (35.4)	<0.001
**High blood pressure**	11,645 (20.7)	15,791 (24.8)	0.004
**Anxiety or related disorder**	8,677 (15.4)	13,929 (21.8)	<0.001
**Feeling depressed**			0.01
A little of the time	9,993 (18.0)	11,296 (17.9)	
All the time	1,209 (2.2)	1,917 (3.0)	
Most of the time	4,600 (8.3)	5,247 (8.3)	
None of the time	31,091 (56.0)	32,163 (51.0)	
Some of the time	8,659 (15.6)	12,414 (19.7)	
**Probable PTSD**	9,557 (17.1)	15,534 (24.7)	<0.001
**Migraine**	7,638 (13.6)	11,017 (17.3)	0.01
**Diabetes**	4,110 (7.3)	5,228 (8.2)	0.3
**Bowel disorders**	3,847 (6.8)	5,547 (8.7)	
**Asthma**	3,540 (6.3)	4,658 (7.3)	0.3
**Heart disease**	2,471 (4.4)	3,581 (5.6)	0.06
**Stomach ulcers**	2,462 (4.4)	3,500 (5.5)	0.2
**Urinary incontinence**	2,259 (4.1)	2,863 (4.5)	0.5
**Traumatic brain injury**	2,314 (4.2)	4,240 (6.8)	0.002
**Cancer**	1,420 (2.5)	1,738 (2.7)	0.7
**COPD**	1,462 (3.1)	2,476 (4.5)	0.07
**Stroke**	559 (1.00)	645 (1.0)	>0.9
Dementia	106 (0.2)	99 (0.2)	0.7
**Mood disorder**	11,680 (20.7)	16,745 (26.3)	<0.001
**Alcohol consumption**			0.6
Every day	4,325 (9.2)	5,323 (10.2)	
Monthly	19,157 (40.6)	20,667 (39.5)	
Weekly	23,658 (50.2)	26,277 (50.3)	

COPD = chronic obstructive pulmonary disease.

^a^Wilcoxon rank sum test for complex survey samples; chi-squared test with Rao & Scott’s second-order correction.

### Factors associated with chronic pain

Our adjusted regression model demonstrated adequate fit, as indicated by a nonsignificant Hosmer-Lemeshow test (*P* = 0.12), and no multicollinearity issues were detected with variance inflation factors for all independent variables below 2. In our adjusted regression model, the year of survey administration was not associated with chronic pain (OR = 1.08; 95% CI 0.55–2.12). Reporting chronic pain was associated with older age (OR for every 3-year increment starting at age 19 = 1.18; 95% CI 1.03–1.35; ARI 2.7%), obesity class 1 (OR = 3.66; 95% CI 1.46–9.17; ARI 27.3%), obesity class 2 (OR = 8.10; 95% CI 1.67–39.3; ARI 46.6%), self-reported mood disorder (OR = 3.20; 95% CI 1.49–6.88; ARI 24.0%), and self-reported anxiety or a related disorder (OR = 4.53; 95% CI 1.28–16.0; ARI 32.6%) (Table 3). When restricted to veteran respondents to the 2016 and 2019 LASS without risk factors for chronic pain (i.e., nonobese, in the lowest age quartile, without self-reported mood disorder or an anxiety or related disorder), the prevalence of chronic pain was 19.8%. This subgroup represented approximately 11% of all veteran respondents. We used this prevalence as the baseline risk to calculate the ARI for all statistically significant risk factors.

**Table 3.** Factors associated with chronic pain.

Characteristic	Adjusted OR	95% CI	*P* value	Absolute risk increase (95% CI)
**Year of survey administration**				
LASS 2016	Reference			
LASS 2019	1.08	0.55, 2.12	0.80	
**Age**				
For every 3-year increment (starting at age 19)	1.18	1.03, 1.35	0.02	2.7% (0.5, 5.1)
**Sex**				
Male	Reference			
Female	1.81	0.52, 6.22	0.30	
**BMI**				
Normal weight	Reference			
Pre-obese	1.45	0.63, 3.31	0.40	
Obese class 1	3.66	1.46, 9.17	0.01	27.3% (6.6, 49.3)
Obese class 2	8.10	1.67, 39.3	0.01	46.6% (9.2, 71.1)
**Mood disorder (self-reported)**				
No	Reference			
Yes	3.20	1.49, 6.88	0.003	24.0% (6.9, 42.8)
**Anxiety or related disorder (self-reported)**				
No	Reference			
Yes	4.53	1.28, 16.0	0.02	32.6% (4.1, 60.0)
**Probable PTSD (self-reported)**				
No	Reference			
Yes	2.21	0.81, 6.02	0.12	
**Alcohol consumption**				
Every day	Reference			
Monthly	1.49	0.44, 5.02	0.7	
Weekly	1.84	0.64, 5.28	0.3	
**Education**				
University graduation	Reference			
High school graduation	0.84	0.29, 2.39	0.7	
Less than high school graduation	3.79	0.60, 23.8	0.2	
Postsecondary graduation	0.58	0.19, 1.75	0.3	
**Household income**				
<50,000	Reference			
≥50,000	0.50	0.20, 1.26	0.14	
**Military rank**				
Junior NCM	Reference			
Officer	0.81	0.24, 2.67	0.7	
Senior NCM	1.10	0.42, 2.87	0.8	
**Military environment**				
Sea	Reference			
Land	1.37	0.56, 3.34	0.5	
Air	1.16	0.45, 2.97	0.8	

## Discussion

The most recent administration of the LASS in 2019 reported a large increase in the prevalence of chronic pain among Canadian veterans from 2016; however, when adjusted for factors associated with chronic pain, the association between year of survey administration and chronic pain became statistically nonsignificant. Our findings suggest that the increased prevalence of chronic pain between the 2016 and 2019 administrations of the LASS is the result of confounding by other factors that are associated with chronic pain. Specifically, compared to veterans who responded to the 2016 LASS, those who completed the 2019 LASS were more likely to present with comorbidities that are associated with chronic pain.

National Canadian data suggest that the prevalence of chronic pain has been relatively stable over time. An analysis of seven cycles of the Canadian Community Health Survey found that the annual prevalence of chronic pain was 16.3% in 2000 and 17.2% in 2010. The authors noted some additional increase in prevalence between 2011 to 2014 but found that increased reporting of chronic pain was largely restricted to younger adults (<29 years) who were otherwise healthy and indicated that their pain was “noninterfering.”^27^

Several prior studies have supported our findings that older age,^17^ obesity,^17,28–30^ and mood and anxiety disorders^17,31^ are associated with chronic pain. However, the cross-sectional nature of LASS data limits our ability to establish causal relationships between these factors and chronic pain. For example, chronic pain may be a cause or a result of obesity and mental illness. Considering the factors that we found associated with chronic pain, there was no statistically significant difference in the prevalence of obesity among veterans who responded to the 2016 and 2019 LASS—in both administrations approximately 30% were obese. Respondents in 2019 were an average of 2.2 years older; however, our analysis indicates that this difference would only be expected to increase the prevalence of chronic pain by about 2%. It is important to acknowledge that some confidence intervals, such as those for obesity class 2, were quite wide, likely due to small cell sizes.

Compared to individuals who completed the 2016 LASS, we observed increases in the prevalence of mood disorders (5.6% risk difference) and an anxiety or related disorder (6.4% risk difference) among veterans that completed the 2019 LASS. In contrast, an analysis of all annual cycles of the Canadian Community Health Survey from 2000 to 2016, including 331,046 adults, found that the prevalence of self-reported mood disorders remained statistically stable over time at 5.4% (95% CI 4.7%–6.0%). This analysis reported a “modest” increase in the annual prevalence of self-reported anxiety disorder (beta coefficient 0.26% among employed participants), but the authors cautioned that prevalence and regression coefficient estimates showed high heterogeneity, suggesting that factors other than time may be influencing trends.^32^ A more recent study enrolled 1412 adults living in Hamilton, Ontario, Canada, and assessed them for symptoms of depression or anxiety ten times from October 2018 to April 2022.^33^ This study found that approximately 85% of individuals showed no change in symptoms over time; approximately 10% reported an increase in symptoms of anxiety and depression and 5% a decrease in symptoms, but most changes were subclinical.

The discrepancy between our findings and population trends suggests that the observed increase in chronic pain prevalence among veterans is a result of changes in the characteristics of survey respondents, particularly regarding mental health comorbidities. This interpretation is supported by a 2023 systematic review of 42 studies found the pooled prevalence of chronic noncancer chronic pain among veterans was 45% but that this estimate was associated with high heterogeneity. Between-study variability was explained, in part, by studies that oversampled veterans with conditions associated with chronic pain (e.g., traumatic brain injury, spinal cord injury, substance use disorder, homelessness, mood disorder). When restricted to studies that enrolled participants representative of a general population of veterans, the review found moderate certainty evidence, as per the GRADE approach,^34^ for an overall pooled prevalence of chronic non-cancer pain of 30% (95% CI 23%–37%).^35^

## Limitations

Our study has several limitations. First, our findings are limited by reliance on self-reported data, which may be subject to recall bias. Second, the question we used to assess the prevalence of chronic pain, “Are you usually free of pain or discomfort?,” lacks specificity regarding pain duration, potentially leading to an overestimation of chronic pain prevalence. Future administrations of the LASS should consider addition of an item to specifically capture chronic pain that has persisted for 3 months or longer. Third, LASS items capturing mental health comorbidities are not consistent with current *Diagnostic and Statistical Manual of Mental Disorders*, Fifth Edition (DSM-5) terminology.^36^ Obsessive compulsive disorder was moved out of the anxiety disorders category in DSM-5 into its own category. Dysthymia is now termed “persistent depressive disorder” and combines dysthymia and chronic major depressive disorder. Further, the LASS questions for PTSD do not establish that there was a Criterion A trauma (i.e., specifically asking whether the person was exposed to death, threatened death, actual or threatened serious injury, or actual or threatened sexual violence). In addition, the criterion that the event causes intense fear, hopelessness, or horror was removed from DSM-5. Finally, the diagnosis of PTSD in DSM-5 has new symptom clusters requiring six symptoms from four symptom clusters. Thus, the LASS no longer conforms to a proxy screen of PTSD, which is why we described respondents who screened positive as presenting with “probable PTSD.” We recommend that these LASS items be revised in future administrations to ensure concordance with DSM-5 language. Fourth, development of our regression model exploring factors associated with chronic pain among Canadian veterans was constrained by data collected by the LASS surveys. As such, several factors that have shown an association with chronic pain (e.g., cultural background, smoking status, physical activity, attitudes and beliefs about pain)^17^ could not be considered. Fifth, due to potential overlap among participants responding to both the 2016 and 2019 administrations of the LASS, we were unable to distinguish and account for individuals participating in multiple survey cycles. This overlap may have resulted in inflated standard errors in our estimates. However, the random selection process and the large target population make substantial overlap unlikely. Finally, our reliance on cross-sectional data restricts our ability to establish causal relationships between chronic pain and associated risk factors.

## Research and policy implications

The 2016 Department of Health and Human Services National Pain Strategy emphasized the need for epidemiological studies of pain, particularly in subpopulations that may be susceptible to underreporting and/or undermanagement of pain,^37^ which includes military veterans.^38^ The LASS is a valuable tool in this regard; however, our findings suggest that the survey is vulnerable to selection bias and may exaggerate the prevalence of chronic pain among Canadian veterans. Efforts to increase the validity of findings from the LASS could include refining the question on chronic pain to explicitly specify a duration of 3 months and revising items designed to capture mental illness to ensure concordance with DSM-5 language. Expanding the scope of data collection to include additional relevant variables, such as ethnicity, physical activity, and attitudes and beliefs about pain, would facilitate more nuanced analyses between chronic pain and risk factors. Further, efforts to further improve the proportion of veterans who are approached and complete the LASS would reduce the survey’s vulnerability to selection bias.

From a policy perspective, our findings support that chronic pain is common among Canadian veterans but also suggest that the prevalence has not appreciably increased over time. As such, though resources to optimize care of Canadians veterans living with chronic pain remain essential, there is unlikely to be additional strain on capacity because of large increases in the prevalence of chronic pain.

## Conclusion

Our analysis of LASS data found that chronic pain among veterans was associated with older age, obesity, self-reported mood disorder, and endorsing an anxiety or related disorder. The higher prevalence of chronic pain among Canadian veterans from 2016 to 2019 appears confounded by increased comorbid mental illnesses associated with chronic pain among responders in 2019.

## Supplementary Material

Manuscript_Veterans and chronic pain_track changes.docx

## Data Availability

The study used data from Statistics Canada’s Life After Service Studies. Access to this data is available upon approval from Statistics Canada. Researchers seeking access should contact Statistics Canada for application and data access guidelines.

## Appendix

**Table A1.** LASS questions for evaluating probable PTSD.

Question number	Question
1	Have you ever had any experience that was so frightening, horrible, or upsetting that, in the past month, you have had nightmares about it or thought about it when you did not want to?
2	Have you ever had any experience that was so frightening, horrible, or upsetting that, in the past month, you tried hard not to think about it or went out of your way to avoid situations that reminded you of it?
3	Have you ever had any experience that was so frightening, horrible, or upsetting that, in the past month, you were constantly on guard, watchful, or easily startled?
4	Have you ever had any experience that was so frightening, horrible, or upsetting that, in the past month, you felt numb or detached from others, activities, or your surroundings?
