# Supplementary material for: An exploration of the increasing prevalence of chronic pain among Canadian veterans: Life After Service Studies 2016 and 2019
Source: Can J Pain. 2025 Jan 30;9(1):2443631. doi: 10.1080/24740527.2024.2443631 (PMC11784647; doi:10.1080/24740527.2024.2443631)
Supplement: Manuscript_Veterans and chronic pain_track changes.docx [file UCJP_A_2443631_SM6685.docx]

**An exploration of the increasing prevalence of chronic pain among Canadian Veterans: Life After Service Studies 2016 and 2019**

Jhalok Ronjan Talukdar^a,b^, Dena Zeraatkar^a,b^, Andrew Thomas^c^, Jason W. Busse^a,b,d^

^a^Department of Anesthesia, McMaster University, Hamilton, Ontario, Canada

^b^Department of Health Research Methods, Evidence, and Impact, McMaster University, Hamilton, Ontario, Canada

^c^ Canadian Armed Forces Health Services Centre, Edmonton, Alberta, Canada

^d^ Michael G DeGroote National Pain Centre, McMaster University, Hamilton, Ontario, Canada

Email addresses:

Jhalok Ronjan Talukdar: talukdaj@mcmaster.ca

Dena Zeraatkar: zeraatd@mcmaster.ca

Andrew Thomas: agthomas5211@hotmail.com**Corresponding Author**

Jason W. Busse

Department of Anesthesia, McMaster University

1280 Main Street West

Hamilton, Ontario, L8S 4K1

Email: bussejw@mcmaster.ca

# Abstract

## Background

The Life After Service Study (LASS) suggests the absolute prevalence of chronic pain among Canadian Veterans, defined as pain lasting three months or longer, increased by 10% from 2016 to 2019.

## Aims

We explored the association of year of survey administration, sociodemographic, military, and health-related factors, with the prevalence of chronic pain among Canadian Veterans.

## Methods

We analyzed 2016 and 2019 LASS data and built a multivariable regression model to explore factors associated with chronic pain. Measures of association are reported as adjusted odds ratios (ORs) and absolute risk increases (ARIs).

***Results***

The 2016 LASS (73% response rate; 3,002 of 4,121) reported a 41.4% prevalence of chronic pain, and the 2019 LASS (72% response rate; 2,630 of 3,671) reported a 51.5% prevalence of chronic pain among Canadian Veterans. Respondents who completed the 2019 LASS were more likely to endorse an anxiety or related disorders, mood disorder, probable post-traumatic stress disorder, and traumatic brain injury. In our adjusted regression model, year of survey administration was not associated with chronic pain (OR 1.08, p=0.8); however, we found large associations with obesity class 1 (BMI 30.0–34.9) (OR 3.66, 95%CI 1.46 to 9.17; ARI 27%), obesity class 2 (BMI 35.0–39.9) (OR 8.10, 95%CI 1.67 to 39.3; ARI 47%), mood disorder (OR 3.20, 95%CI 1.49 to 6.88; ARI 24%), and an anxiety or related disorder (OR 4.53, 95%CI 1.28 to 16.0; ARI 33%).

## Conclusions

The increase in chronic pain among Canadian Veterans from 2016 to 2019 appears confounded by increased co-morbidities associated with chronic pain among responders in 2019.

**Keywords:** Chronic pain, Life After Service Study, Chronic pain among Canadian Veterans, Canadian Armed Forces, cross-sectional survey

**Full-text word count:** 2,749

# Introduction

Chronic pain, which persists beyond the normal time required for healing and is commonly defined as pain lasting ≥3 months, impacts approximately 20% of the general population in Canada, the USA, and globally ^1-7^. In Canada, two-thirds of people living with chronic pain experience moderate to severe symptoms, and half report living with chronic pain for over a decade ^2^. Chronic pain is not evenly distributed among the Canadian population, with a higher prevalence reported among older adults, Indigenous peoples, women, Veterans, and people who use drugs ^2^.

Chronic pain affects Veterans' well-being, with 54% reporting pain interference with work and 77% experiencing life stress most days ^8^. There is also a strong association between chronic pain and mental health and 63% of Veterans living with chronic pain meet diagnostic criteria for a mental illness^8^. Several factors may contribute to the higher rates of chronic pain among Veterans. These include the physically demanding nature of military service, a higher prevalence of mental health conditions compared to the general population, combat-related injuries, potential delays in accessing appropriate pain management during the transition from military to civilian healthcare systems, and cultural factors such as military emphasis on resilience that may lead to delayed reporting of pain. ^8-12^

The Life After Service Study (LASS), conducted by Statistics Canada every three years, is a national survey that collects information from military Veterans. According to results from the LASS, the percentage of Canadian Veterans reporting chronic pain increased by 10.1% over the two most recent administrations, from 41.4% in 2016 ^6^ to 51.5% in 2019 ^13^. The underlying factors for this increase remain unclear; however, we hypothesized that contributing factors may include older age or confounding by other factors associated with chronic pain. Specifically, Veterans who responded to the LASS in 2019 may have been systematically different than those who responded in 2016.

# Materials and methods

## Study design and participants

The LASS is a national cross-sectional telephone survey that collects comprehensive data on Canadian Armed Forces (CAF) Regular Force Veterans, including their health, well-being, and demographic factors ^14^. The 2016 LASS surveyed Veterans discharged from the CAF between 1998 and 2015, and the 2019 LASS surveyed Veterans discharged between 1998 and 2018. Eligible respondents for both surveys were CAF members who were not currently enlisted, resided in Canada's 10 provinces, were not in a long-term care facility, and did not hold entry rank at discharge.^14,15^ We included 3,002 participants (73% response rate; 3,002 of 4,121)^16^ from the 2016 LASS (representing a weighted total population of 56,413) and 2,630 participants (72% response rate; 2,630 of 3,671)^17^ from the 2019 LASS (representing a weighted total population of 63,948) in our analysis. The same Veterans could potentially participate in multiple cycles of the LASS; however, the data does not allow for identification of common respondents between administrations.

## Variables

### Chronic Pain

We classified participants as having chronic pain if they endorsed the following item on the 2016 or 2019 LASS survey: "Are you usually free of pain or discomfort?" (Yes or No). While the descriptor “usually” implies a temporal aspect, it is not clear if Veterans who responded in the affirmative to this question had experienced pain for 3 months or longer. Nevertheless, we accepted this definition of chronic pain to maintain consistency with previous analyses of LASS data ^6,17^ reporting on the prevalence of chronic pain, to facilitate direct comparisons ^13,18^. If a respondent endorsed living with chronic pain on the LASS, then pain intensity and activity limitations were captured through the following questions: 1) “How would you describe the usual intensity of your pain or discomfort?” (mild, moderate, or severe); and 2) “How many activities does your pain or discomfort prevent?” (none, a few, some, or most).

### Covariates

We captured data on 11 covariates to include in our regression model: age at the time of survey completion; sex (male or female); formal education (less than high school graduation, high school graduation, post-secondary graduation, or university graduation); annual household income (< $50,000 or ≥ $50,000); military rank at discharge (Junior Non-Commissioned Members, Officer, or Senior Non-Commissioned Members); military environment (sea, land, or air); body mass index [BMI] (underweight: BMI <18.5, normal: BMI 18.5–24.9, pre-obese: BMI 25.0–29.9, obese-class 1: BMI 30.0–34.9, obese-class 2: BMI 35.0–39.9, obese-class 3: BMI >40); alcohol consumption (everyday, weekly, monthly); self-reported mood disorder (e.g., major depressive disorder, persistent depressive disorder, bipolar disorder) (yes or no); an anxiety or related disorder (e.g., phobia, obsessive compulsive disorder, panic disorder) (yes or no); and probable posttraumatic stress disorder (PTSD) (yes or no). Regarding mental health conditions, the LASS surveys assess anxiety or related disorders and mood disorders by asking participants whether they are experiencing each of these conditions. These questions specifically inquire about conditions diagnosed by a health professional that are expected to last, or that have already lasted, 6 months or more. The LASS assesses probable PTSD if respondents endorse at least 3 of 4 questions about this disorder (see Appendix Table 1).

Our selection of covariates was informed by prior research demonstrating positive associations between older age, female sex, higher BMI, mental illness, lower formal education attainment and lower household income with chronic pain. ^11,19-22^ Lower military rank may entail greater exposure to physical and psychological stressors, which can increase the risk of chronic pain ^23,24^. Similarly, military environment (sea, land, or air) may influence the development of chronic pain, with land-based roles often involving more physical strain and higher risk of injury ^25^. Further, a 2022 systematic review found that any alcohol consumption was associated with a 24% lower odds of reporting chronic pain ^26^.

## Statistical analysis

We present descriptive statistics to summarize the characteristics of participants and the distribution of chronic pain among Canadian Veterans between surveys, using weighted estimations. We report categorical variables as frequencies and percentages and continuous variables as means and standard deviations (SD) when normally distributed and as medians with interquartile ranges otherwise. We used the Wilcoxon rank-sum test for complex survey samples for continuous variables and the chi-squared test with Rao & Scott’s second-order correction, accounting for survey design, for categorical variables, to compare responses between 2016 and 2019. Bootstrap weights provided by Statistics Canada were applied to convert unweighted frequencies to represent the Canadian Veteran population ^16^.

Using weighted data from both the 2016 and 2019 LASS surveys, we constructed a multivariable logistic regression model to identify factors associated with chronic pain among Canadian Veterans. Our independent factors were: (1) year of survey administration; (2) age; (3) sex; (4) formal education; (5) household income; (6) military rank; (7) military environment; (8) BMI; (9) alcohol consumption; (10) self-reported mood disorder; (11) self-reported anxiety or a related disorder; and (12) probable PTSD. To avoid overfitting, we required 10 or more events per variable included in our regression model ^27^. We excluded response options that were endorsed by less than 3% of respondents. We reported relative measures of association as odds ratios (ORs) and 95% confidence intervals (95% CIs). Multicollinearity was deemed concerning if the variance inflation factor for any independent variable was greater than five ^28^. All comparisons were 2-tailed, and an independent factor was considered statistically significant if it had a p-value < 0.05 in our multivariable model. For all statistically significant factors, we calculated the absolute risk increase (ARI) to optimize interpretability. We established the baseline risk for reporting chronic pain by calculating the prevalence among those Veteran respondents without statistically significant risk factors for chronic pain. We used the Hosmer–Lemeshow test ^29^ to assess the goodness-of-fit of our logistic regression model, and conducted all analyses with R version 4.3.3 (Vienna, Austria) ^30^.

# Results

Respondents to the 2016 and 2019 LASS surveys were similar in sex distribution, BMI, marital status, household income, military rank and environment. Approximately 87% of Veterans were men, 62% were married, more than 80% reported household incomes of ≥ $50,000 per year, and most held a Junior Non-Commissioned Members miliary rank at discharge. Respondents in 2019 were an average of 2.2 years older (SD 0.07), more likely to have completed post-secondary education (41% v. 36%), and less likely to be employed (59% v. 66%) than respondents in 2016 (Table 1).

Compared to 2016, Veterans who completed the LASS in 2019 were more likely to report chronic pain (41.4% v. 51.5%); pain severity and activity limitations among those with chronic pain were similar between administrations. Consistent with the higher prevalence of chronic pain, respondents to the 2019 LASS also reported higher prevalences of pain-related conditions, including back problems (46% v. 41%), arthritis (35% v. 30%) and migraine headaches (17% v. 14%). Veterans who completed the 2019 LASS were also more likely to self-report an anxiety or related disorder (22% v. 15%), a mood disorder (49% v. 44%), probable PTSD (25% v. 17%), and traumatic brain injury (7% v. 4%) (Table 2).

## Factors associated with chronic pain

Our adjusted regression model demonstrated adequate fit, as indicated by a non-significant Hosmer-Lemeshow test (p = 0.12), and no multicollinearity issues were detected with variance inflation factors for all independent variables below 2. In our adjusted regression model, the year of survey administration was not associated with chronic pain (OR, 1.08; 95%CI, 0.55 to 2.12). Reporting chronic pain was associated with older age (OR for every 3-year increment starting at age 19, 1.18; 95%CI, 1.03 to 1.35; ARI, 2.7%), obesity class 1 (OR, 3.66; 95%CI, 1.46 to 9.17; ARI, 27.3%), obesity class 2 (OR, 8.10; 95%CI, 1.67 to 39.3; ARI, 46.6%), self-reported mood disorder (OR, 3.20; 95%CI, 1.49 to 6.88; ARI, 24.0%), and self-reported anxiety or a related disorder (OR, 4.53; 95%CI, 1.28 to 16.0; ARI, 32.6%). When restricted to Veteran respondents to the 2016 and 2019 LASS without risk factors for chronic pain (i.e., non-obese, in the lowest age quartile, without self-reported mood disorder or an anxiety or related disorder), the prevalence of chronic pain was 19.8%. This subgroup represented approximately 11% of all Veteran respondents. We used this prevalence as the baseline risk to calculate the ARI for all statistically significant risk factors.

# Discussion

The most recent administration of the LASS in 2019 reported a large increase in the prevalence of chronic pain among Canadian Veterans from 2016; however, when adjusted for factors associated with chronic pain the association between year of survey administration and chronic pain became statistically non-significant. Our findings suggest that the increased prevalence of chronic pain between the 2016 and 2019 administration of the LASS is the result of confounding by other factors that are associated with chronic pain. Specifically, compared to Veterans who responded to the 2016 LASS, those who completed the 2019 LASS were more likely to present with comorbidities that are associated with chronic pain.

National Canadian data suggests the prevalence of chronic pain has been relatively stable over time. An analysis of seven cycles of the Canadian Community Health found the annual prevalence of chronic pain was 16.3% in 2000 and 17.2% in 2010. The authors noted some additional increase in prevalence between 2011 to 2014 but found that increased reporting of chronic pain was largely restricted to younger adults (<29 years) who were otherwise healthy and indicated their pain was “noninterfering” ^31^.

Several prior studies have supported our findings that older age,^19^ obesity, ^19,32-34^ mood and anxiety disorders ^19,35^ are associated with chronic pain. However, the cross-sectional nature of LASS data limits our ability to establish causal relationships between these factors and chronic pain. For example, chronic pain may be a cause or a result of obesity and mental illness. Considering the factors that we found associated with chronic pain, there was no statistically significant difference in the prevalence of obesity among Veterans who responded to the 2016 and 2019 LASS – in both administrations approximately 30% were obese. Respondents in 2019 were an average of 2.2 years older; however, our analysis indicates that this difference would only be expected to increase the prevalence of chronic pain by about 2%. It is important to acknowledge that some confidence intervals, such as those for obesity class 2, were quite wide, likely due to small cell sizes.

Compared to individuals who completed the 2016 LASS, we observed increases in the prevalence of mood disorders (5% risk difference) and an anxiety or related disorder (7% risk difference) among Veterans that completed the 2019 LASS. In contrast, an analysis of all annual cycles of the Canadian Community Health Survey from 2000 to 2016, including 331,046 adults, found the prevalence of self-reported mood disorders remained statistically stable over time at 5.4% (95% CI 4.7% to 6.0%). This analysis reported a “modest” increase in the annual prevalence of self-reported anxiety disorder (beta-coefficient 0.26%, among employed participants) but the authors cautioned that prevalence and regression coefficient estimates showed high heterogeneity suggesting that factors other than time may be influencing trends ^36^. A more recent study enrolled 1,412 adults living in Hamilton, Ontario, Canada, and assessed them for symptoms of depression or anxiety ten times from October 2018 to April 2022 ^37^. This study found that approximately 85% of individuals showed no change in symptoms over time; approximately 10% reported an increase in symptoms of anxiety and depression, and 5% a decrease in symptoms, most changes were subclinical.

The discrepancy between our findings and population trends suggests that the observed increase in chronic pain prevalence among Veterans is a result of changes in the characteristics of survey respondents, particularly regarding mental health comorbidities. This interpretation is supported by a 2023 systematic review of 42 studies found the pooled prevalence of chronic noncancer chronic pain among Veterans was 45%, but that this estimate was associated with high heterogeneity. Between-study variability was explained, in part, by studies that oversampled Veterans with conditions associated with chronic pain (e.g., traumatic brain injury, spinal cord injury, substance use disorder, homelessness, mood disorder). When restricted to studies that enrolled participants representative of a general population of Veterans, the review found moderate certainty evidence, as per the GRADE approach ^38^, for an overall pooled prevalence of chronic non-cancer pain of 30% (95% CI 23% to 37%) ^39^.

## Limitations

Our study has several limitations. First, our findings are limited by reliance on self-reported data, which may be subject to recall bias. Second, the question we used to assess the prevalence of chronic pain, "Are you usually free of pain or discomfort?" lacks specificity regarding pain duration, potentially leading to an overestimation of chronic pain prevalence. Future administrations of the LASS should consider addition of an item to specifically capture chronic pain that has persisted for 3 months or longer. Third, LASS items capturing mental health comorbidities are not consistent with current DSM-5 terminology. Obsessive compulsive disorder was moved out of the anxiety disorders category in DSM-5 into its’ own category. Dysthymia is now termed “persistent depressive disorder” and combines dysthymia and chronic major depressive disorder. Further, the LASS questions for PTSD do not establish that there was a Criterion A trauma (i.e., specifically asking if the person was exposed to death, threatened death, actual or threatened serious injury, or actual or threatened sexual violence). In addition, the criterion that the event causes intense fear, hopelessness, or horror was removed from DSM 5. Finally, the diagnosis of PTSD in DSM 5 has new symptom clusters requiring 6 symptoms from 4 symptom clusters. Thus, the LASS no longer conforms to a proxy screen of PTSD, which is why we described respondents who screened positive as presenting with “probable PTSD”. We recommend that these LASS items be revised in future administrations to ensure concordance with DSM-5 language. Fourth, development of our regression model exploring factors associated with chronic pain among Canadian Veterans was constrained by data collected by the LASS surveys. As such, several factors which have shown an association with chronic pain (e.g., cultural background, smoking status, physical activity, attitudes and beliefs about pain) ^19^ could not be considered. Fifth, due to potential overlap among participants responding to both the 2016 and 2019 administrations of the LASS, we were unable to distinguish and account for individuals participating in multiple survey cycles. This overlap may have resulted in inflated standard errors in our estimates. However, the random selection process and the large target population make substantial overlap unlikely. Finally, our reliance on cross-sectional data restricts our ability to establish causal relationships between chronic pain and associated risk factors.

## Research and policy implications

The 2016 Department of Health and Human Services National Pain Strategy emphasised the need for epidemiological studies of pain, particularly in subpopulations that may be susceptible to under-reporting and/or undermanagement of pain ^40^, which includes military Veterans ^41^. The LASS is a valuable tool in this regard; however, our findings suggest that the survey is vulnerable to selection bias and may exaggerate the prevalence of chronic pain among Canadian Veterans. Efforts to increase the validity of findings from the LASS could include refining the question on chronic pain to explicitly specify a duration of 3 months and revising items designed to capture mental illness to ensure concordance with DSM-5 language. Expanding the scope of data collection to include additional relevant variables, such as ethnicity, physical activity, and attitudes and beliefs about pain would facilitate more nuanced analyses between chronic pain and risk factors. Further, efforts to further improve the proportion of Veterans that are approached and complete the LASS would reduce the survey's vulnerability to selection bias.

From a policy perspective, our findings support that chronic pain is common among Canadian Veterans but also suggest that the prevalence has not appreciably increased over time. As such, while resources to optimize care of Canadians Veterans living with chronic pain remain essential, there is unlikely to be additional strain on capacity because of large increases in the prevalence of chronic pain.

# Conclusion

Our analysis of LASS data found that chronic pain among Veterans was associated with older age, obesity, self-reported mood disorder, and endorsing an anxiety or related disorder. The higher prevalence of chronic pain among Canadian Veterans from 2016 to 2019 appears confounded by increased co-morbid mental illnesses associated with chronic pain among responders in 2019.

# Abbreviations

BMI: body mass index

CAF: Canadian Armed Forces

COPD: chronic obstructive pulmonary disease

LASS: Life After Service Study

PTSD: posttraumatic stress disorder

TBI: traumatic brain injury

# Acknowledgments

The authors are grateful to the Canadian Armed Forces members who participated in the LASS surveys, and Dr. Peter Kitchen (Statistics Canada Research Data Centre at McMaster University) for statistical support. We also thank Dr. Randi McCabe (Professor, Psychiatry & Behavioural Neurosciences, McMaster University) for reviewing questions from the Life After Service Study (LASS) designed to identify mental health issues to reconcile them with current DSM-5 language.

# Ethical approval

We obtained ethical approval for our study from the Hamilton Integrated Research Ethics Board (HiREB), REB #17136.

# Informed consent

The Life After Service Study (LASS) data were obtained through the Statistics Canada Research Data Centre at McMaster University. Statistics Canada conducted the survey using Computer Assisted Telephone Interviews (CATI). Prior to data collection, introductory letters were sent to potential participants with available mailing addresses. At the start of each telephone interview, interviewers provided a standardized introduction explaining the purpose of the survey and how results would be used. Participants were explicitly informed that their participation was voluntary and assured that their information would remain confidential. This process ensured that informed consent was obtained from all participants before proceeding with the survey.

# Funding

This study was funded by the Chronic Pain Centre of Excellence for Canadian Veterans. Jason Busse is supported, in part, by a CIHR Research Chair in the prevention and management of chronic pain.

# Role of funder

The funding organization was not involved in any aspect of the study, including its design, conduct, manuscript preparation, review, approval, or submission for publication.

# Disclosure statement

The authors have no conflicts of interest to disclose.

# Contributions

Conceptualization: JWB, DZ, AT and JRT

Methodology: JWB, DZ and JRT

Writing – Original draft: JRT

Writing – Review and Editing: JWB, DZ, AT and JRT

# ORCID

# Jhalok Ronjan Talukdar: https://orcid.org/0000-0001-9739-5632

# Dena Zeraatkar: https://orcid.org/0000-0003-4287-0541

# Jason W. Busse: https://orcid.org/0000-0002-0178-8712

Andrew Thomas: https://orcid.org/0000-0001-5494-9623

# Data availability

# The study used data from Statistics Canada's Life After Service Studies. Access to this data is available upon approval from Statistics Canada. Researchers seeking access should contact Statistics Canada for application and data access guidelines.

# References

1. Treede RD, Rief W, Barke A, Aziz Q, Bennett MI, Benoliel R, Cohen M, Evers S, Finnerup NB, First MB, et al. A classification of chronic pain for ICD-11. Pain 2015;156(6):1003-7.

2. Canadian Pain Task Force. Chronic pain in Canada: laying a foundation for action: A report by the Canadian Pain Task Force*.* Ottawa, ON: Health Canada;2019.

3. Cohen SP, Vase L, Hooten WM. Chronic pain: an update on burden, best practices, and new advances. The Lancet 2021;397(10289):2082-97.

4. Dahlhamer J, Lucas J, Zelaya C, Nahin R, Mackey S, DeBar L, Kerns R, Von Korff M, Porter L, Helmick C. Prevalence of Chronic Pain and High-Impact Chronic Pain Among Adults - United States, 2016. MMWR Morb Mortal Wkly Rep 2018;67(36):1001-6.

5. Jiang Y, Xu T, Mao F, Miao Y, Liu B, Xu L, Li L, Sternbach N, Zhou M, Fan B. The prevalence and management of chronic pain in the Chinese population: findings from the China Pain Health Index (2020). Population Health Metrics 2022;20(1):20.

6. Linda Van Til D, Sweet J, Poirier A, McKinnon K, Pedlar D, Sudom K, Dursun S. Well-being of Canadian regular force Veterans, findings from LASS 2016 survey*.* Veterans Affairs Canada Charlottetown (PE); 2017.

7. Rikard SM, Strahan AE, Schmit KM, Guy Jr GP. Chronic pain among adults—United States, 2019–2021. Morbidity and Mortality Weekly Report 2023;72(15):379.

8. Statistics Canada. Chronic Pain in Veterans. 2024, at <https://www.veterans.gc.ca/en/about-vac/research/info-briefs/chronic-pain-veterans>).

9. Chronic Pain Centre of Excellence for Canadian Veterans. Understanding Chronic Pain For Veterans & Families. 2024. (Accessed September 12, 2024, at <https://www.veteranschronicpain.ca/veterans>).

10. May K, Sud A, Redburn D. Canadian Armed Forces Veterans living with chronic pain. Canadian Medical Association Journal 2024;196(23):E792.

11. VanDenKerkhof EG, VanTil L, Thompson JM, Sweet J, Hopman WM, Carley ME, Sudom K. Pain in Canadian Veterans: analysis of data from the Survey on Transition to Civilian Life. Pain Res Manag 2015;20(2):89-95.

12. Ul Haq MZ, Ashoorion V, Xi CE, Wang E, Ross N, Parakh N, Busse JW, Darzi AJ, Alvarez E. Canadian Veterans’ Experiences of Living with Chronic Pain: A Descriptive Qualitative Study. Canadian Journal of Pain 2024;8(2):2361006.

13. Nazari G, Reyes-Vélez J, Thompson JM, Sweet J, Miller J. Well-being of Veterans with chronic pain with fewer activities limited by pain: Life After Service Survey 2019. Journal of Military, Veteran and Family Health 2023;9(3):70-85.

14. Statistics Canada. Life After Service Survey (LASS): Detailed information for 2019. 2020. (Accessed March 15, 2024, at <https://www23.statcan.gc.ca/imdb/p2SV.pl?Function=getSurvey&Id=1228312>).

15. Statistics Canada. Life After Service Survey (LASS): Detailed information for 2016 2017. (Accessed March 15, 2024, at <https://www23.statcan.gc.ca/imdb/p2SV.pl?Function=getSurvey&Id=257809>).

16. Van Til LD, Sweet J, Poirier A, McKinnon K, Sudom K, Dursun S, Pedlar D. Well-being of Canadian Regular Force Veterans, findings from the LASS 2016 survey*.* Veterans Affairs Canada Charlottetown, PE; 2017.

17. Sweet J, Poirier A, Pound T, Van Til L. Well-being of Canadian regular force Veterans, findings from LASS 2019 survey*.* Veterans Affairs Canada Charlottetown (PE); 2020.

18. Van Til LD, Sweet J, Poirier A, McKinnon K, Sudom K, Dursun S, Pedlar D. Well-being of Canadian Regular Force Veterans, findings from the LASS 2016 survey. 2017, at <http://publications.gc.ca/pub?id=9.839366&sl=0>).

19. Mills SEE, Nicolson KP, Smith BH. Chronic pain: a review of its epidemiology and associated factors in population-based studies. Br J Anaesth 2019;123(2):e273-e83.

20. van Hecke O, Torrance N, Smith BH. Chronic pain epidemiology and its clinical relevance. BJA: British Journal of Anaesthesia 2013;111(1):13-8.

21. van Hecke O, Torrance N, Smith BH. Chronic pain epidemiology - where do lifestyle factors fit in? Br J Pain 2013;7(4):209-17.

22. van Hecke O, Torrance N, Smith BH. Chronic pain epidemiology and its clinical relevance. Br J Anaesth 2013;111(1):13-8.

23. Reyes Velez J, Thompson JM, Sweet J, Busse JW, VanTil L. Cluster analysis of Canadian Armed Forces veterans living with chronic pain: Life After Service Studies 2016. Can J Pain 2021;5(1):81-95.

24. Schoenfeld AJ, Cirillo MN, Gong J, Bryan MR, Banaag A, Weissman JS, Koehlmoos TP. Development of Chronic Pain Conditions Among Women in the Military Health System. JAMA Network Open 2024;7(7):e2420393-e.

25. Schoenfeld AJ, Cirillo MN, Gong J, Bryan MR, Banaag A, Weissman JS, Koehlmoos TP. Development of Chronic Pain Conditions Among Women in the Military Health System. JAMA Netw Open 2024;7(7):e2420393.

26. Karimi R, Mallah N, Nedjat S, Beasley MJ, Takkouche B. Association between alcohol consumption and chronic pain: a systematic review and meta-analysis. Br J Anaesth 2022;129(3):355-65.

27. Harrell FE. Multivariable Modeling Strategies. Regression Modeling Strategies: With Applications to Linear Models, Logistic Regression and Survival Analysis New York, NY: Springer; 2001:53-85.

28. Belsley DA, Kuh E, Welsch RE. Regression Diagnostics: Identifying Influential Data and Sources of Collinearity.: John Wiley & Sons; 1980.

29. Fagerland MW, Hosmer DW. A generalized Hosmer–Lemeshow goodness-of-fit test for multinomial logistic regression models. The Stata Journal 2012;12(3):447-53.

30. R Core Team. R: A language and environment for statistical computing. 2020, at <https://www.R-project.org/>).

31. Shupler MS, Kramer JK, Cragg JJ, Jutzeler CR, Whitehurst DGT. Pan-Canadian Estimates of Chronic Pain Prevalence From 2000 to 2014: A Repeated Cross-Sectional Survey Analysis. The Journal of Pain 2019;20(5):557-65.

32. Nilsen TIL, Holtermann A, Mork PJ. Physical Exercise, Body Mass Index, and Risk of Chronic Pain in the Low Back and Neck/Shoulders: Longitudinal Data From the Nord-Trøndelag Health Study. American Journal of Epidemiology 2011;174(3):267-73.

33. Wright LJ, Schur E, Noonan C, Ahumada S, Buchwald D, Afari N. Chronic Pain, Overweight, and Obesity: Findings from a Community-Based Twin Registry. The Journal of Pain 2010;11(7):628-35.

34. Stokes AC, Xie W, Lundberg DJ, Hempstead K, Zajacova A, Zimmer Z, Glei DA, Meara E, Preston SH. Increases in BMI and chronic pain for US adults in midlife, 1992 to 2016. SSM - Population Health 2020;12:100644.

35. Perera E, Thompson JM, Asmundson GJ, El-Gabalawy R, Afifi TO, Sareen J, Bolton S-L. Chronic pain: The Canadian Armed Forces members and Veterans mental health follow-up survey. Journal of Military, Veteran and Family Health 2021;7(S2):29-42.

36. Dobson KG, Vigod SN, Mustard C, Smith PM. Trends in the prevalence of depression and anxiety disorders among working-age Canadian adults between 2000 and 2016. Health Rep 2020;31(12):12-23.

37. Scarfe ML, Belisario K, Gillard J, De Jesus J, Frey BN, Van Ameringen M, McKinnon MC, Gohari MR, Busse JW, MacKillop J. Periodicity and severity of changes in depression and anxiety during the COVID-19 pandemic: Ten-wave longitudinal findings from an observational cohort study of community adults. Psychiatry Res 2023;326:115267.

38. Guyatt G, Oxman AD, Akl EA, Kunz R, Vist G, Brozek J, Norris S, Falck-Ytter Y, Glasziou P, deBeer H, et al. GRADE guidelines: 1. Introduction—GRADE evidence profiles and summary of findings tables. Journal of Clinical Epidemiology 2011;64(4):383-94.

39. Qureshi AR, Patel M, Neumark S, Wang L, Couban RJ, Sadeghirad B, Bengizi A, Busse JW. Prevalence of chronic non-cancer pain among military veterans: a systematic review and meta-analysis of observational studies. BMJ Mil Health 2023.

40. Committee IPRC. National pain strategy: a comprehensive population health-level strategy for pain. Washington, DC: US Department of Health and Human Services, National Institutes of Health 2016;36.

41. Patel M, Jomy J, Couban RJ, Scelleur HL, Busse JW. Transition Needs Among Veterans Living With Chronic Pain: A Systematic Review. Mil Med 2024;189(3-4):556-65.
